# Open-Source Algorithm for Automated Vigilance State Classification Using Single-Channel Electroencephalogram in Rodents

**DOI:** 10.3390/s25030921

**Published:** 2025-02-03

**Authors:** Anton Saevskiy, Natalia Suntsova, Peter Kosenko, Md Noor Alam, Andrey Kostin

**Affiliations:** 1 Scientific Research and Technology Center for Neurotechnology, Southern Federal University, 344006 Rostov-on-Don, Russia; saevskiy@sfedu.ru (A.S.); pokosenko@sfedu.ru (P.K.); 2 Research Service (151A3), Veterans Affairs Greater Los Angeles Healthcare System, Sepulveda, Los Angeles, CA 91343, USA; suntsova@ucla.edu; 3 Department of Medicine, David Geffen School of Medicine, University of California, Los Angeles, CA 90095, USA

**Keywords:** single-channel EEG signal, automatic sleep stage classification, GMM clustering, NREM sleep, REM sleep

## Abstract

Accurate identification of sleep stages is essential for understanding sleep physiology and its role in neurological and behavioral research. Manual scoring of polysomnographic data, while reliable, is time-intensive and prone to variability. This study presents a novel Python-based algorithm for automated vigilance state scoring using single-channel electroencephalogram (EEG) recordings from rats and mice. The algorithm employs artifact processing, multi-band frequency analysis, and Gaussian mixture model (GMM)-based clustering to classify wakefulness, non-rapid, and rapid eye movement sleep (NREM and REM sleep, respectively). Combining narrow and broad frequency bands across the delta, theta, and sigma ranges, it uses a majority voting system to enhance accuracy, with tailored preprocessing and voting criteria improving REM detection. Validation on datasets from 10 rats and 10 mice under standard conditions showed sleep–wake state detection accuracies of 92% and 93%, respectively, closely matching manual scoring and comparable to existing methods. REM sleep detection accuracies of 89% (mice) and 91% (rats) align with previously reported (85–90%). Processing a full day of EEG data within several minutes, the algorithm is advantageous for large-scale and longitudinal studies. Its open-source design, flexibility, and scalability make it a robust, efficient tool for automated rodent sleep scoring, advancing research in standard experimental conditions, including aging and sleep deprivation.

## 1. Introduction

### 1.1. Background and Context

The relationship between physiological and pathological conditions and the sleep–wake cycle has been extensively studied in both humans and laboratory animals, particularly rodents. These studies often require continuous polysomnographic (PSG) recordings to accurately identify sleep–wake states. In rodents, PSG typically includes electroencephalogram (EEG) and electromyogram (EMG) channels, which provide sufficient information to detect three main vigilance states: wakefulness, non-rapid eye movement (NREM) sleep, and rapid eye movement (REM) sleep.

Traditionally, PSG signal analysis for sleep staging is performed manually, a process that is labor-intensive and time-consuming, especially in longitudinal studies spanning weeks or months. Moreover, the accuracy of manual scoring varies between different experts and even within the same scorer over time, achieving an agreement rate of 85–95% [1,2,3,4,5]. To address these limitations, various automated scoring algorithms have been developed [6]. Automated methods enable rapid analysis of extensive long-term EEG recordings and exhibit robustness against inconsistencies in ambiguous vigilance states, which are prone to errors during manual analysis.

### 1.2. Current Approaches and Limitations

Similar to manual scoring, automated algorithms for rodents typically rely on both EEG and EMG recordings, with EMG believed to enhance the detection of short arousals and REM sleep. However, our experience and reports from other studies [7] suggest that EMG quality tends to degrade over time in longitudinal experiments. This deterioration affects the accuracy of sleep–wake identification and may lead to the exclusion of animals with severely contaminated or weakened EMG signals from the analysis.

Recent studies have demonstrated that a single EEG channel, without an accompanying EMG channel, can provide sufficient information to differentiate between wakefulness, NREM, and REM sleep [7,8,9,10]. Reducing the number of PSG channels is advantageous for long-term experiments as it simplifies data acquisition and minimizes trauma to the animals. However, the choice of EEG derivation can influence the accuracy of vigilance state detection, prompting standardization of optimal cortical recording sites in both rats and mice [7,11].

The most basic approaches to automatic EEG analysis employ traditional signal processing and statistical methods, involving filtering, feature extraction, and statistical measures like mean, variance, or spectral power [4,12]. These methods are interpretable and do not rely heavily on labeled training data. However, they may be less flexible and struggle to capture complex relationships in EEG data compared to more advanced methods like artificial neural networks (ANNs) or other machine learning algorithms. A notable limitation of traditional methods is the requirement for manual configuration of parameters and thresholds, which can introduce variability between different users and even within the same user over time.

While ANN-based approaches for analyzing EEG data have demonstrated potential superior efficiency [13,14,15,16,17], these methods require substantial amounts of expert-annotated data for training and have high computational demands. These requirements currently limit their adoption in many sleep research laboratories. Additionally, once trained, ANN models may not generalize well to new, previously unseen examples, necessitating extensive model fine-adjusting.

Another class of methods includes clustering-based approaches [7,18,19,20], which are a subtype of machine learning that does not require predefined category labels. Recent reports indicate that these methods can effectively separate NREM sleep from wakefulness and REM sleep, although perfect separation of REM sleep from other states remains challenging, especially when REM sleep identification relies on high-frequency theta activity [6,7,9,21].

### 1.3. Challenges in Sleep Staging

The main challenge arises from a relatively small proportion of REM sleep compared to NREM sleep and wakefulness, coupled with the similarity of its EEG patterns to those observed during wakefulness. Therefore, achieving clear separation of REM sleep from wakefulness by applying clustering methods in a straightforward manner is challenging, as the REM sleep cluster often significantly overlaps with the wake cluster, and to a lesser degree, with NREM clusters. Additionally, most clustering-based approaches still require expert oversight to validate cluster quality and may struggle when EEG features are highly specific due to the distinct characteristics of the animal group under study or technical limitations. It is of great methodological importance to develop an approach that integrates traditional clustering with unique preprocessing techniques, resulting in a novel methodology that enhances the robustness of identification, particularly during transitional epochs.

### 1.4. Innovative Aspects of the Approach Used in This Study

Our approach to vigilance state classification combines traditional clustering with original preprocessing steps, followed by a series of refinement procedures to achieve accurate epoch classification. Specifically, we minimized the risk of misclassifying REM sleep as high-frequency theta wakefulness by implementing a preprocessing step that removes a significant portion of wakefulness epochs while preserving the majority of REM sleep epochs. This step is crucial for the subsequent analysis as it significantly enhances the separation of REM sleep from other states, reducing false positives for both REM sleep and wake epochs.

Instead of using broad EEG frequency ranges, we optimized stage classification accuracy by identifying the most effective combinations of multiple narrow bands, tailored to species-specific sleep patterns. For behavioral state identification, we applied the GMM clustering algorithm to each frequency band and employed a novel voting procedure for final classification, where the predominant classification across multiple bands determined the stage of each epoch.

We developed and validated a user-friendly, Python-based algorithm for scoring vigilance states in rodents using single-channel EEG data. Our approach integrates traditional signal processing, statistical analysis, and clustering techniques, prioritizing transparency and ease of implementation for researchers. Notably, our method does not require a training set; it is designed to capture the natural structure of the sleep–wake cycle, enabling the analysis of new data without calibration while maintaining accuracy comparable to manual scoring. All parameters of the algorithm are adjustable, allowing for optimization during exploratory analysis when a manually scored reference dataset is available. Additionally, the software includes interactive tools for manual parameter adjustments, further enhancing accuracy and performance.

Many existing sleep-scoring algorithms have been developed and validated using data from a single rodent species, restricting their applicability to broader models. In contrast, our study assessed algorithm performance in both rats and mice, demonstrating its adaptability and robustness across species.

Our automatic scoring algorithm was evaluated against conventional visual analysis by expert scorers, achieving a high overall accuracy of 0.92 in rats and 0.93 in mice, comparable to manual scoring. These findings validate the generalizability and reliability of our automated approach.

## 2. Materials and Methods

### 2.1. Animal Dataset

The polysomnographic recordings, consisting of one EEG and one EMG signal per animal, were obtained from previous studies conducted in our laboratory [22,23] and included the following:

i. Ten EEG recordings from Fischer344 male rats aged 4–12 months that included periods of spontaneous sleep–wake behavior or 6 h of sleep deprivation followed by recovery, with a combined total duration of 216 h (Table 1).
ii. Ten EEG recordings from C57BL/6 mice of both genders aged 5 and 25 months, with a total duration of 240 h of spontaneous sleep–waking recording from undisturbed animals (Table 1).

In this study, mouse EEG recordings were used to illustrate the algorithm’s performance steps, preventing redundancy from similar diagrams for rats. However, species-specific results of the study were presented separately for both rats and mice.

During data collection, animals were maintained on a 12:12 light: dark cycle, with an ambient temperature of 24 ± 2 °C. Food and water were available ad libitum throughout the study. All experiments were conducted in accordance with the National Research Council’s “Guide for the Care and Use of Laboratory Animals” and were approved by the Institutional Animal Research Committee of the Veterans Affairs Greater Los Angeles Healthcare System.

### 2.2. Surgery and Data Acquisition

The details of the surgical procedures have been described previously [24,25]. Briefly, under anesthesia (Ketamine + Xylazine: 80:10 mg/kg; i.p.) and aseptic conditions, EEG electrodes were implanted for unilateral bipolar recording from the parietal cortex with the following stereotaxic coordinates for rostral and caudal electrodes, respectively:

i. For mice, AP: 1 mm and −3 to −4 mm, and LM+: 1 mm and 2 mm;
ii. For rats, AP: 1 mm and −6 mm, and LM:1 mm and 3 mm.
algorithm.

Following recovery and adaptation, amplified EEG and EMG signals were continuously recorded at sampling rates of 104 Hz and 256 Hz, respectively, and stored in .SMR format.

### 2.3. Manual Annotation

All EEG data from rats and mice were annotated by the same experienced scorer. For that, ten-second epochs of polysomnographic recordings were visually annotated using Spike2 (CED electronics, Cambridge, UK) software into three vigilance states: wake, NREM sleep, and REM sleep, according to the conventional criteria [6,11,26].

The information about vigilance states was converted into a hypnogram and stored in separate *.txt files with the same name as corresponding *.SMR file. For automatic annotation, only a single EEG derivation was used.

### 2.4. Automatic Annotation Algorithm Based on a Single EEG Channel

In the following sections, user-adjustable parameters of the sleep scoring algorithm are highlighted using italic bold font. The algorithm is implemented as a Python-based Jupyter Notebook file, allowing stepwise code block execution [27].

Users can open digital polysomnographic files with various extensions supported by the Neo library for Python [28]. To do so, the user needs to define the required reader class from the library.

**Figure 1:**
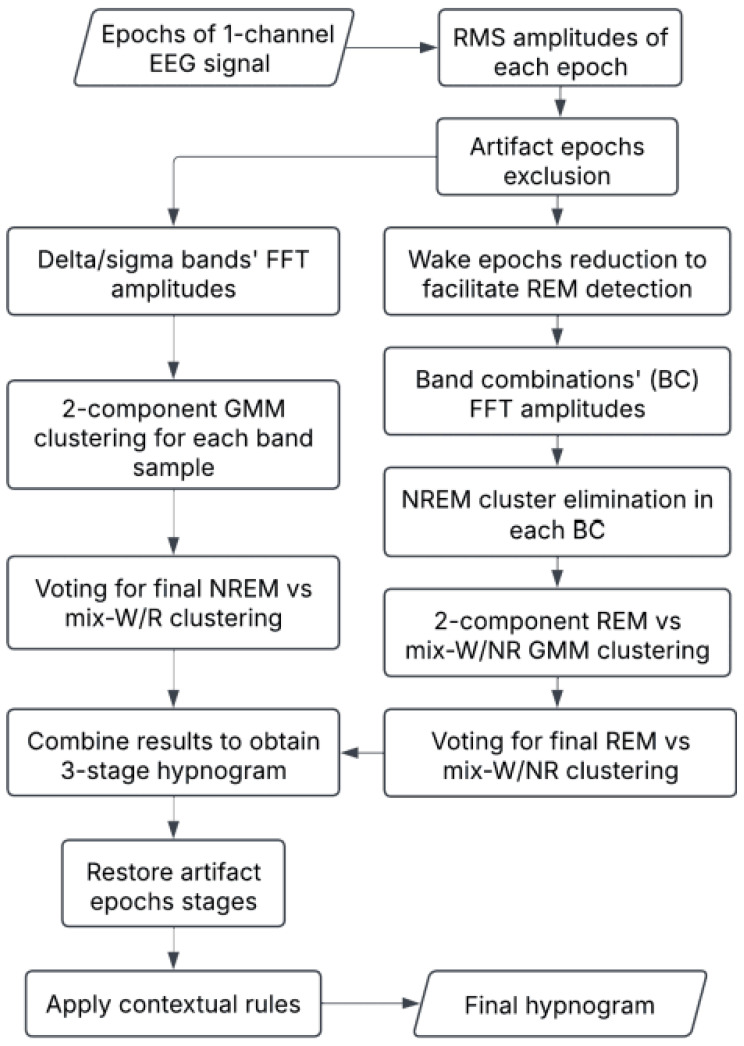
The flowchart of the suggested automatic vigilance state scoring.

The developed algorithm is summarized in the block diagram (Figure 1), and all the steps involved are described in detail in the following sections.

To ensure optimal functionality and reproducibility, the following Python software packages and libraries were employed:

Holoviews (1.17.1); hvplot (0.8.4); jupyterlab (3.6.3); matplotlib (3.6.2); mne (1.2.3); neo (0.11.1); numpy (1.23.5); pandas (1.5.2); scikit-learn (1.2.0); and scipy (1.9.3)

This implementation ensures compatibility with a wide range of data formats and computational environments, providing researchers with a flexible, open-source tool for sleep scoring in rodent models.

### 2.5. General Preprocessing

If the sampling rate was non-integer, a correction procedure is applied to round it to the nearest integer value. This ensures more precise splitting of the signal into epochs with integer lengths in both points and seconds. This procedure is implemented in the resampling function of the MNE library for Python [29,30]. The resulting data are then divided into epochs of a user-defined length in seconds, denoted as array X.

To reduce artifacts, whenever necessary, a high-pass FIR filter was applied with a cutoff frequency of 2 Hz, a transition width of 0.6 Hz, and a filter length determined based on the sampling rate (typically 8 × sampling period).

### 2.6. Artifact Detection

Correct identification of sleep–wake states requires the exclusion of epochs contaminated with artifacts. Two types of artifacts were detected: (1) high-amplitude artifacts exceeding 4 standard deviations (SD) above the mean RMS value, and (2) normal-amplitude artifacts identified using kernel density estimation with Epanechnikov’s kernel, using a density threshold of −5 (z-score normalization).

### 2.7. High-Amplitude Artifact Identification

i. Transforming EEG signal to RMS amplitudes (root mean square). To extract the information on the overall amplitudes of epochs, we use the RMS series computed over the epochs of the raw signal *X* [31].
We used a 10-s analysis window; however, a window length ranging from 2 to 30 s can be employed if necessary [32].
ii. Smoothing. Smoothing of the raw RMS signal is required to reduce amplitude variations. The smoothing window of 20 s (extending 10 s to the left and 10 s to the right of each RMS series point) was established empirically, and the *X^RMS^* series was transformed according to the following formula:

(1) $$X_{n}^{\prime RMS}=\sqrt{\frac{1}{w+1}\;\sum_{j=n-w}^{n+w}{\left(X_{j}^{RMS}\right)}^{2}}$$

where *j* is the epoch index and *w* is the smoothing window length.

iii. Fitting the Gaussian mixture model (GMM). Fitting a 2-component Gaussian mixture model (GMM) to the smoothed data is performed to detect epochs with high and low RMS amplitudes. The Gaussian mixture model (GMM) is a standard clustering method that decomposes the data into a predefined number of components (each assumed to follow a Gaussian distribution) to provide the most optimal and realistic centroids and covariance matrices [33,34]. Both components may be contaminated with artifacts: epochs with ~0 amplitudes assigned to the lower component may indicate signal absence, and the high-amplitude outliers assigned to the higher component typically represent movement artifacts.
iv. Threshold-based automatic artifact identification. Two thresholds for the removal of both low- and high-amplitude artifacts are then calculated according to the following formulas:

$$T_{high}=M_{high}+4\cdot \sigma_{high}$$

$$T_{low}=M_{low}-4\cdot \sigma_{low}$$

(subscripts “*high*” and “*low*” denote the GMM components found; *T* is the threshold, *M* is the mean value, and *σ* is the standard deviation).

**Figure 2:**
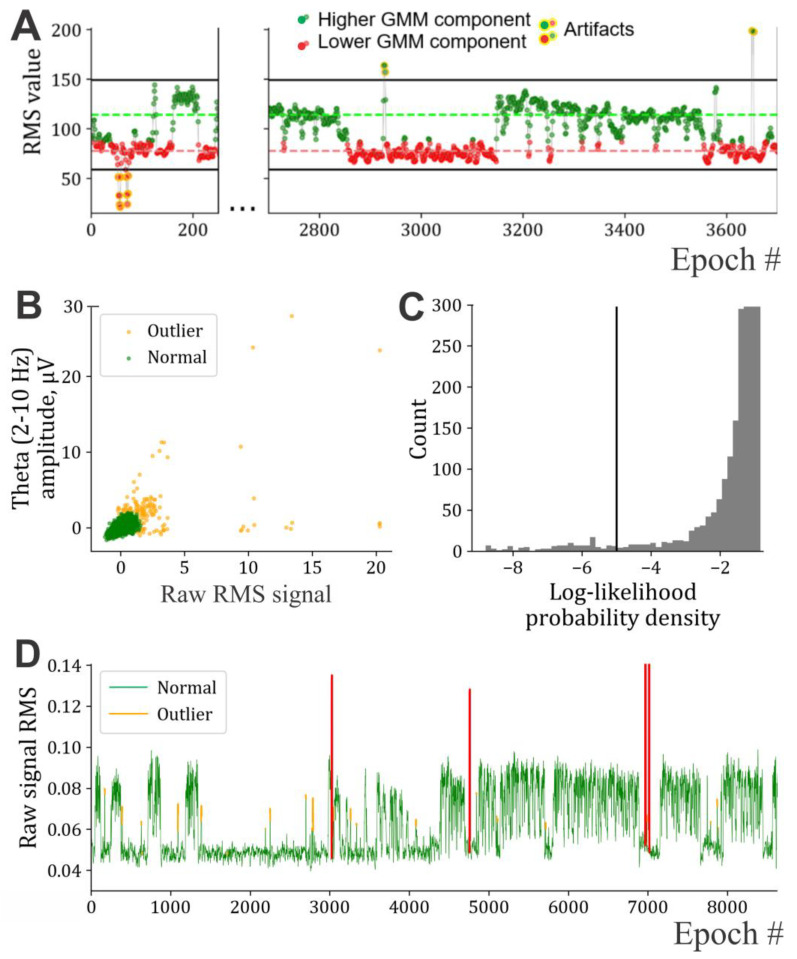
Identification and processing of artifacts. (**A**). Dotted lines are the mean values of the corresponding components, and the resulting thresholds are shown as black lines; points corresponding to identified artifact epochs are outlined with orange. (**B**–**D**). Detection of the artifacts with amplitudes within the normal range of values for 24-h mouse data; (**B**) scatter plot of raw signal RMS and 2–10 Hz band FFT amplitude feature space, with kernel density estimation results color-coded based on the threshold from (**C**); (**C**) histogram of log-likelihood probability density values based on the points from (**B**); (**D**) one-dimensional representation of the raw signal RMS, highlighting the identified outliers (red chunks were identified using the thresholds from (**A**) and the orange ones correspond to the results of (**B**,**C**)).

Epochs with amplitudes falling outside the range defined by the thresholds are considered artifact epochs and are excluded from further analysis (Figure 2A). If the suggested method fails (it rarely occurs when the number and/or extent of outliers is too large), thresholds can be set manually using visual criteria.

### 2.8. Low-Amplitude Artifacts Detection

After the initial step of reducing artifacts with abnormal amplitudes, some artifacts with amplitudes within the normal range of values may remain (Figure 2B–D). To detect and exclude epochs contaminated with these artifacts, the following procedures are performed:

i. FFT is applied to each 10-s epoch using a 256-point Hanning window with 50% overlap:

$$X\left(f\right)=\sum_{n=0}^{N-1}X(n)e^{\frac{-2i\pi nf}{N}}$$

ii. Creating a two-dimensional sample where the first dimension is the RMS of the raw signal used previously for finding artifact thresholds, and the second is the series of summed FFT-spectrum amplitudes in the 2–10 Hz frequency range;
iii. Transforming these dimensions to their standard scores, i.e., z-scores;
iv. In the obtained 2-dimensional feature space, most of the points form a Gaussian-like distribution, while the artifact points of interest are outliers (Figure 2B). These outliers are automatically identified using kernel density estimation with Epanechnikov’s kernel [34]. The method numerically assesses what is visually apparent in the scatter plot (some points are closer together) by evaluating the density of data points at every location in the feature space. This allows for distinguishing areas based on data point density. Since the values were previously z-scored, the density threshold was empirically determined to be −5 regardless of the data (Figure 2C). Figure 2D shows an example of when both types of artifacts are detected.

The classification of the excluded from analysis artifact epochs was made after the final hypnogram was acquired. The epochs were attributed to the stage of the closest preceding non-artifact epochs.

### 2.9. Splitting the Multiclass Stage Scoring into Binary Problems

Identification of major vigilance states based solely on analysis of EEG signal is challenging because two states, wakefulness and REM sleep, exhibit very similar EEG characteristics.

To address this problem, we first performed specific preprocessing and transformations of the EEG signal. We then approached the classification of sleep–wake stages into three classes by breaking it down into simpler binary classification tasks: (a) distinguishing NREM sleep from a mixture of wakefulness and REM sleep, and (b) distinguishing REM sleep from a mixture of wakefulness and NREM sleep. Solving the two binary classification problems for the three classes allows us to identify the remaining epochs as representing the third class (wake).

### 2.10. NREM Sleep Detection

The electrographic hallmarks of NREM sleep are high-amplitude delta waves (1–4 Hz) and sleep spindles—bursts of oscillatory activity within the sigma frequency range (11–16 Hz). Delta and sigma bands were selected to distinguish NREM sleep from the mixture of wakefulness and REM sleep (mix-W/R) because the amplitude of oscillations within these bands is greater during NREM sleep compared to W and REM sleep [35].

In existing automatic scoring algorithms, each EEG epoch is typically assigned based on conventional frequency bands associated with NREM sleep (1–4 Hz or 11–16 Hz) or wakefulness and REM sleep (5–9 Hz) [6], although their borders are approximate and conditional. Since the amplitude-frequency characteristics of EEG are highly dynamic, utilizing multiple frequency bands within the delta and sigma ranges offers more detailed information about each epoch. This approach can enhance the accuracy of state identification, particularly for ambiguous epochs. Moreover, optimizing the frequency band also improves robustness to species- and subject-specific differences. For example, it is known that sigma oscillations during NREM in mice depending on the electrode location may be observed at lower frequencies about 10 Hz [36].

**Figure 3:**
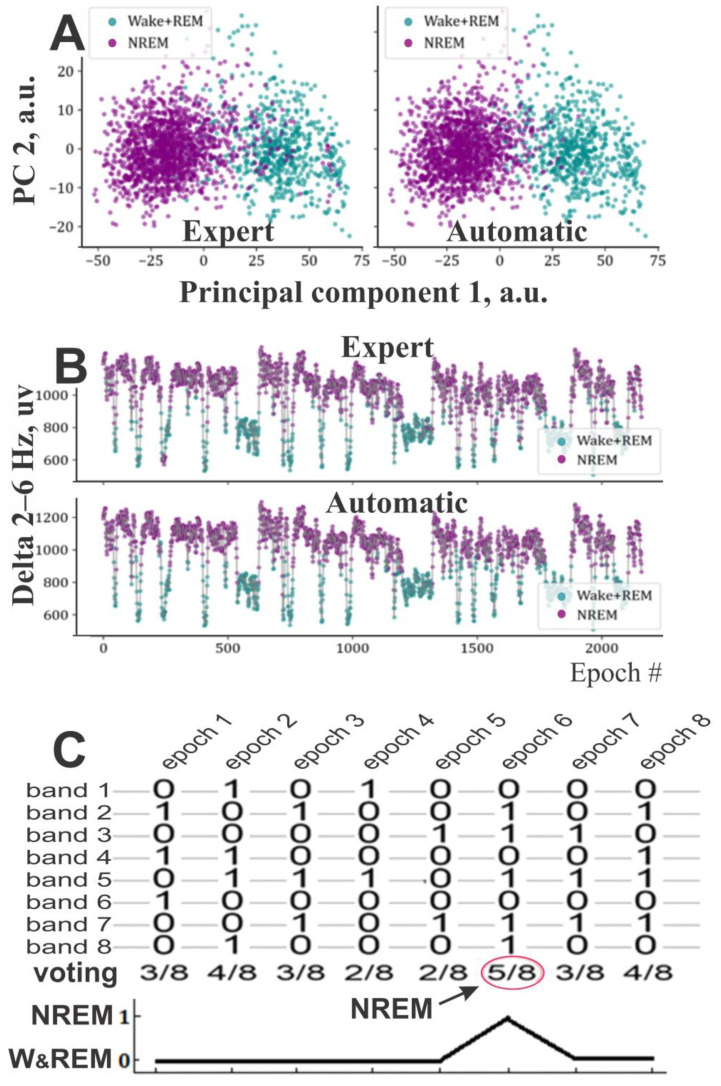
GMM clustering and majority voting for NREM sleep state classification. (**A**). Examples of GMM clustering results obtained for the same 6 h sample of mouse data subjected to manual and automatic scoring. Since the sample included multiple raw FFT frequencies, a 2-dimensional PCA representation of the sample from a single narrow band (2–6 Hz) is plotted for better visualization. (**B**). One-dimensional temporal representation of the same data used in (**A**) (upper and lower plots are color-coded according to the expert and automatic scoring, respectively). (**C**). An illustration of majority “voting” in a binary classification task: NREM sleep (1) vs. W/REM sleep mixture (0). The rows represent 8 frequency bands, and the columns represent 8 ten-second EEG epochs. At the bottom, the resulting pseudo-hypnogram is shown.

In the present study, we evaluated this approach by assessing the efficiency of individual narrow bands and their combinations in distinguishing NREM sleep from mixed wakefulness and REM sleep using GMM. An efficiency chart was constructed based on these assessments (see Results Section). A band or combination was deemed “efficient” if it achieved sensitivities of ≥0.9 for both states. The pipeline for identifying NREM epochs is as follows:

i. FFT features extraction. The FFT amplitudes for the set of delta and sigma bands were extracted from the previously calculated complete FFT spectrum X(f) and stored separately.
ii. Smoothing. Optionally, 2D arrays of the spectra chunks with dimensions ‘epoch number’ and ’frequency’ are RMS-smoothed along the time dimension using a user-defined window length in seconds, as specified in Formula (1). The smoothing reduces excessive fragmentation in the resulting hypnogram and improves the results [11].
iii. GMM clustering. The FFT spectrum values for each frequency band and epoch, organized in a 2D array with dimensions ‘epoch number’ and ‘frequency’, were input into a GMM model to distinguish two components (Figure 3A,B). The identified components were labeled as 1 or 0, with 1 representing NREM sleep and 0 representing a mix of wakefulness and REM sleep. Frequency spectrum chunks corresponding to each band were used without summing or averaging them. This resulted in a higher dimensional feature vector and allowed the GMM to better separate the components. The results of this clustering for a single band are shown in Figure 3A,B. To examine frequency variations across multiple bands, we tracked the locations of specific epochs within GMM clusters generated using different frequency ranges. This approach allows us to observe how these epochs distribute across clusters, providing insight into the variation of frequencies within the defined bands (Supplementary Figure S1A). For visualization purposes, we used the first 2 principal components [37] of the band’s sample, as the raw sample is much higher-dimensional and cannot be plotted directly.
Compared to the conventional approach that uses one-dimensional series and rigid thresholds, the presented method is more effective at detecting ambiguous states. As shown in Figure 3B, a rigid threshold applied to one-dimensional data cannot achieve comparable separation.
iv. Voting. After GMM clustering, each frequency band independently classified epochs as “1” (NREM sleep). Due to the transitional nature of some epochs (Supplementary Figure S1A), discrepancies were expected between classifications across different bands, particularly for epochs near cluster boundaries. To resolve these inconsistencies, a “voting” method was applied across all frequency bands (Figure 3C). An epoch was assigned to NREM sleep when most frequency bands indicated this stage; otherwise, it was retained in the “mix-W/R” group for additional refinement (Figure 3C).

### 2.11. Separation of REM Sleep

**Figure 4:**
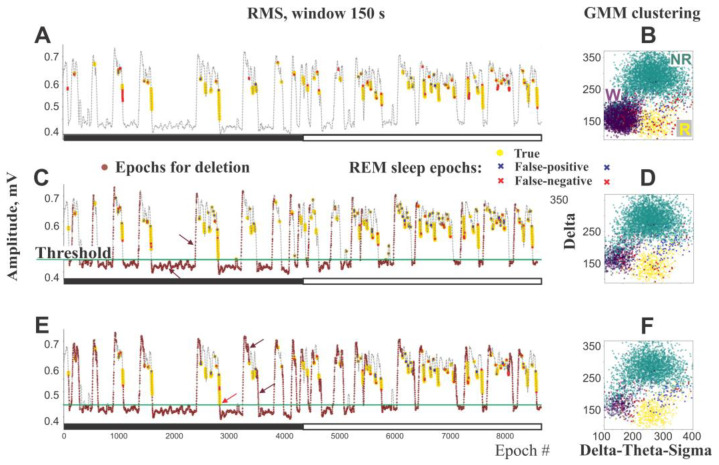
The impact of iterative removal of wake and NREM sleep epochs on REM sleep identification. (**A**,**B**). REM sleep identification without removal wake and NREM sleep epochs. Note dense wake cluster overlapping with REM sleep cluster and numerous false-positive and false-negative REM sleep epochs. (**C**–**F**). REM sleep identification after the first and second iterations of wake/NREM epochs’ elimination, respectively. Brown arrows point to examples of RMS segments that are excluded from the analysis. The red arrow highlights REM sleep epochs located too close to the threshold level, indicating a risk of potential epoch loss and suggesting the need for threshold adjustment. GMM clustering results show a stepwise reduction in false-positive and false-negative REM sleep epochs after each iteration, compared to conditions where wake and NREM sleep epochs are not removed.

Distinguishing REM sleep from a mixture of wakefulness and NREM sleep using a single EEG channel is challenging, as REM sleep exhibits similar EEG characteristics to wakefulness: low amplitude desynchronized activity and prominent theta oscillations. Moreover, the separation of these states using GMM is not sufficiently effective, as evidenced by numerous false positive and false negative REM sleep epochs (Figure 4A). This inefficiency arises due to the relatively small number of REM sleep epochs compared to other states and their substantial overlap with wakefulness epochs (Figure 4B). To overcome the above-mentioned challenges, we suggest the REM sleep identification as a separate step allowing reconsidering previously annotated epochs even if they were previously marked as NREM sleep. Additionally, we suggest some preprocessing steps that significantly facilitate identification of REM sleep. We assume that reducing the number of wake epochs and some NREM sleep epochs in GMM clustering significantly decreases the number of false-positive REM sleep epochs by making the percentages of REM sleep and wake epochs more balanced.

### 2.12. Reducing the Number of Wake Epochs

i. ${{\;X}^{\prime }}^{RMS}$ series was further smoothed using a wide window (w = 150–300 s), according to Formula (1).
ii. Next, to separate low and high values in the smoothed RMS series we applied a 2-component GMM to the RMS. The lower values exclusively contained wake epochs, while the higher values contained NREM sleep, REM sleep, and a small number of wake epochs. Because REM sleep follows NREM sleep immediately, previous smoothing flattens RMS signal disabling its sharp decrease that is typical for NREM-to-REM sleep transitions. After smoothing, most REM sleep epochs occur in the higher component, while most wake epochs remain at the low values level and are rejected. The boundary between the higher and lower components is marked as the threshold (Figure 4C). To ensure all REM sleep epochs are in the higher component, we further decrease the threshold separating the components by 10% of the difference between components’ mean values. Therefore, all lower values, mostly wake epochs, are eliminated, and only the higher values were used for subsequent analysis.
iii. The effect of window length on the removal of wake epochs
Our findings indicate that when the window size is set to 150–300 s and combined with an automatic threshold setting, the retention of REM sleep epochs exceeds 95% and approaches a plateau, while the percentage of wake epochs is reduced by over 80% compared to the prior preprocessing condition (Figure 4A–D). Shorter windows (150–200 s) allow the removal of more wake epochs than wider windows (300 s) with the same threshold but require a stricter threshold setting due to the risk of losing some REM sleep epochs if the threshold is set too high. Therefore, shorter windows are recommended when using a manual threshold. In contrast, in the case of wider windows (≥300 s), the threshold could be more flexible. The wide window approach is more suitable for automatic threshold settings, as it minimizes the loss of REM sleep.
Further increases in window length do not enhance the retention of REM sleep epochs; instead, they lead to a rise in undesirable wake epochs. Therefore, we consider a window length of ~150–300 s to be optimal for both rats and mice.
Further elimination of post-threshold epochs within rising RMS slopes (Figure 4C) allows for the additional removal of wake and NREM sleep epochs while still minimally affecting REM sleep. This process further enhances the accuracy of REM sleep identification due to “cleaning up” the REM sleep cluster from other states, thereby enhancing the performance of the GMM approach for REM sleep identification (Figure 4D).
iv. Removing epochs positioned on the ascending RMS slope after the threshold crossing (Figure 4C) leads to significant further reductions in the number of wakefulness and NREM sleep epochs. Our analysis showed that eliminating sets of 40–50 epochs after the threshold crossing did not impact the number of REM sleep epochs.
v. Additional elimination of wake and NREM sleep epochs is performed during the second cycle of analysis, referred to as the second iteration depicted as i = 1 in Figure 1, which can be optionally executed after completing all steps of the primary analysis, termed the first iteration (Figure 1, i = 0). During this stage, epochs situated on both the ascending and descending slopes of the RMS, positioned between identified REM sleep and the threshold, are deleted (Figure 4E). This process further reduces the size of the wake and NREM sleep clusters (Figure 4F), thereby making the clustering process more efficient for the initial separating and further refining REM sleep classification.

The diagrams display the RMS of 24 h mouse EEGs (A, C, and E), smoothed by using a 150s window, and the corresponding GMM clustering of wake (W), NREM (NR) sleep, and REM (R) sleep (B, D, and F). Yellow depicts REM sleep epochs automatically identified and confirmed by expert scoring. Brown depicts epochs deleted from subsequent analysis. Blue and red mark false-positive and false-negative REM sleep epochs, respectively.

Upon the completion of scoring, we plotted the smoothed RMS, the threshold line, and the automatically identified REM sleep epochs. The proximity of some REM sleep epochs to the threshold line suggests that some epochs could be mistakenly eliminated along with wake epochs (Figure 4E). If too many such epochs are identified, the threshold needs to be adjusted.

### 2.13. Final REM Sleep Identification

**Figure 5:**
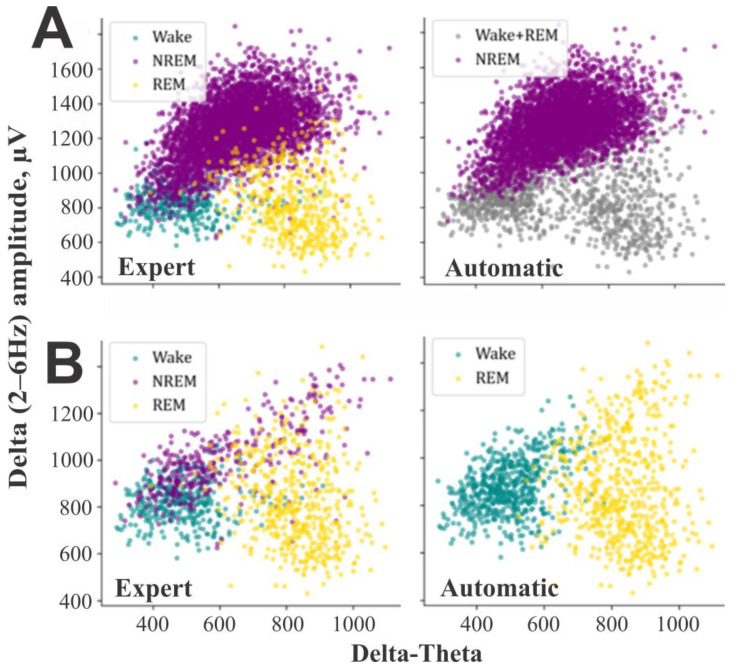
GMM clustering for REM sleep classification. (**A**). An example of GMM clustering representing wakefulness, NREM, and REM sleep according to expert scoring (left) and GMM clustering results in the delta–theta feature space aimed at separating NREM sleep epochs from a mixture of REM sleep and wake epochs (right). (**B**). An example of GMM clustering colored according to expert scoring after removing NREM sleep epochs (left) and GMM clustering applied to the theta component in the delta–theta feature space, aimed at separating a mixture of REM sleep from wake epochs (right).

Multiple 2–4 Hz wide frequency bands, “theta bands”, in the range from 5 to 10 Hz were used to identify REM sleep. Specifically, we used previously computed FFT spectra X(f) for all epochs and obtained new theta features by cutting and summing the spectral chunks corresponding to each separate band. To increase the feature space dimensionality, we complemented each theta band with different delta/sigma bands, referring to these as “theta vs. delta/sigma” band combinations (BCs). Only the epochs attributed to the higher component defined in the previous step were considered for each BC (Figure 5A).

After the elimination of most wake epochs, the remaining epochs still represent a mixture of all three stages. This mixture is more optimally balanced, meaning that the number of wake and REM sleep epochs are comparable, and their clusters are more distinguishable from each other. In the delta-theta feature space, once most wake epochs are removed, the NREM sleep cluster becomes the largest and most easily separable (Figure 5A). Following a stepwise binary classification approach, a two-component GMM is applied to the delta portion of the sample, expecting the NREM sleep cluster to roughly align with the component exhibiting higher delta values. This step aims to minimize the theta–delta sample size while retaining as many REM sleep epochs as possible. Consequently, perfect NREM sleep elimination, which would require complex calculations, is not needed. An example of the results of this step is shown in Figure 5A, where only the grey points are further considered for the next stage of REM sleep identification.

In the remaining wake–REM sleep mixture cluster, the samples of both states are balanced much better than before preprocessing, facilitating further clustering. The REM sleep cluster is typically located either to the right of the wake subcluster, sharing comparable or slightly wider delta/sigma amplitudes but exhibiting higher theta amplitudes than the wake state (Figure 5B). Next, another automatic 2-component GMM clustering is applied to the theta component of the feature space, following the same approach described for delta bands in the ‘NREM-vs-mix-W/R clustering’. The rightmost component is identified as containing REM sleep epochs (Figure 5B).

### 2.14. Revealing EEG Frequency Parameters for Efficient REM Sleep Identification via Clustering

**Figure 6:**
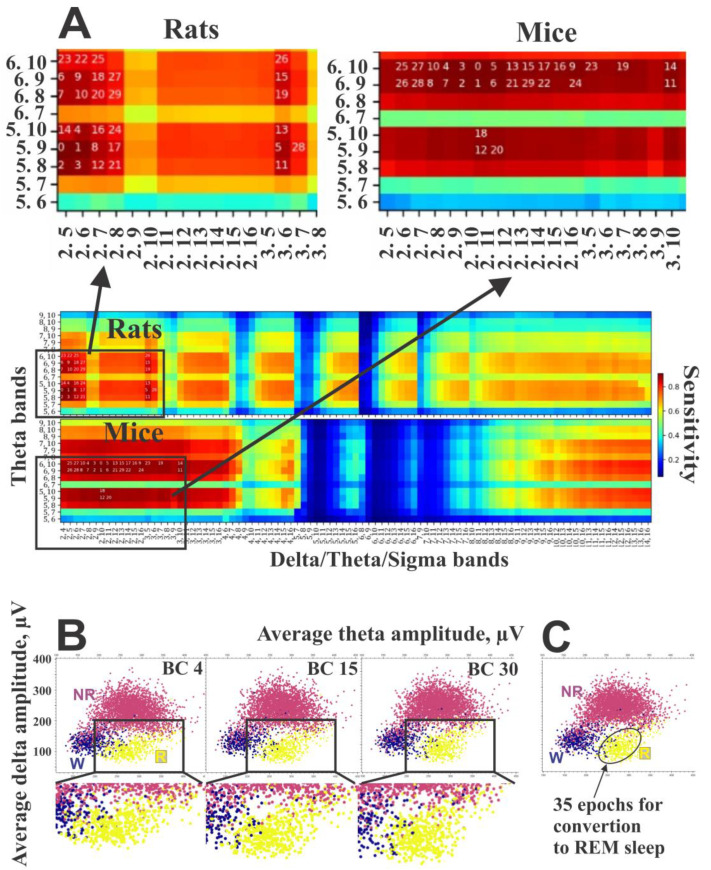
The impact of BC selection on REM sleep classification. (**A**). Sensitivity measures obtained for REM sleep-vs-mix-W/NREM classification, averaged across 6 rats (upper panel) and 9 mice (lower panel) for 1365 BCs. Numbers show the 30 most efficient BCs in decreasing order. (**B**). An example of average theta–delta/sigma clustering based on 4, 15, and 30 BCs demonstrating the role of the BC number on the isolation of REM sleep cluster from wake and NREM sleep clusters. Abbreviations for clusters are the same as in Figure 4. (**C**). An example of average theta–delta/sigma clustering and automatic REM sleep impurities correction approach with standard deviation ellipse magnified 1.5×. All 35 REM sleep epochs misidentified as NREM sleep and wake and located inside the ellipse will be converted into REM sleep epochs.

We designed an approach to identify reliable markers for the robust separation of REM sleep. Our method assumes that REM sleep is characterized by maximal EEG power within a frequency range of 5–10 Hz, coupled with minimal power in the sigma and delta frequencies. The efficiency of the method was tested on 24 h EEG records from 10 rats and 10 mice that were manually scored. We tested pairwise combinations of 91 delta/sigma bands and 15 theta bands, with 1365 paired combinations in total (Table 2). After testing, we selected thirty BCs that demonstrated maximal efficiency in identifying REM sleep. To assess efficiency, “the sensitivity” was employed as a criterion, and the results of all tested BCs were presented on a corresponding heat map for both rats and mice (Figure 6A).

### 2.15. Ambiguity in REM Sleep Epochs Identification

Most REM sleep epochs consistently reside within the REM sleep cluster, regardless of the BC used. These epochs are referred to as “unambiguous”. The unstable REM sleep epochs that appeared around the border between clusters or either in the wake or NREM sleep cluster depending on BC (Supplementary Figure S1B), are referred to as “ambiguous”. The ambiguity of the epoch was defined when a set of “*n”* BCs is used, and a presumable REM sleep epoch resides within the REM sleep clusters a specific number of times (*stringency*) and *n-stringency* number of times in other clusters. After clustering all BCs, the stringency of each epoch was defined, and the status of each epoch was temporally defined as “0” or “2” via the voting using the stringency as the voting threshold. The “2” is a consensual mask of REM sleep. Overall, the voting process for identifying REM sleep epochs followed a similar approach to that used for NREM sleep classification. An epoch was designated as REM sleep if the majority of BCs classified it as such; otherwise, it remained in the mixed group of wake and NREM sleep epochs. However, it is unknown what stringency threshold is optimal within BCs to reliably identify the epoch as REM sleep.

### 2.16. Evaluating the Optimal Number of BCs

Increasing the number of BCs enhances the distinction of the average REM sleep cluster from wake and NREM sleep clusters (Figure 6B), suggesting improved REM sleep identification. To determine the optimal BC count and stringency thresholds, we tested BC numbers ranging from 2 to 30. Results are presented in heat maps (Supplementary Figure S2) indicating accuracy, general F1-score, and REM F1-score based on numbers of BCs and stringencies.

### 2.17. Inversion of False Negative REM Sleep Epochs by Contouring Typical REM Sleep Range

The automatically identified REM sleep cluster usually includes a notable proportion of epochs classified as false negatives. Specifically, the percentage of false negative REM sleep epochs residing within the REM sleep cluster can be as high as 10–30%, while the number of true “wake” or “NREM” epochs located in the same area is significantly lower (Figure 6C). To address this issue, we propose an approach that allows switching between automatic and manual methods.

The automatic method involves selecting the area based on the standard deviation ellipse of the REM sleep cluster. We revealed that the maximal stringency (i.e., 80–100% of the maximal value) produces a REM sleep cluster closely aligning with the cluster of REM sleep epochs annotated based on manual scoring. The ellipse is derived from this cluster. Specifically, a single component of the Gaussian model is fitted, and the ellipsis axes are derived from the component’s covariance matrix (Figure 6C). This ellipsis can be optionally magnified. We empirically determined that the optimal values for this parameter for rats and mice range from 1 to 1.5. Notably, we observed that the quality of automatic REM sleep cluster contouring tends to deteriorate with an increase in artifacts in the EEG data. We recommend that users review the resulting plots of the clusters and the automatic contouring. If users are not satisfied with the automatic results, the manual contouring option is available, offering greater flexibility but requiring some experience in assessing REM sleep clusters. The manual approach involves manually drawing a closed curve [38] within which all epochs are classified as REM sleep.

After the post-processing steps, some wake epochs may still be incorrectly identified as REM sleep, typically accounting for no more than 0.3–3% of total REM sleep across different animals. However, since REM sleep is the least frequent sleep stage, it is important to correct these errors.

### 2.18. Hypnogram Correction via Contextual Rules

At this stage the intermediate hypnogram is generated after applying several contextual rules to eliminate some false-positive REM sleep epochs, reclassifying them to a more probable vigilance state. Indeed, these rules improve the results and are frequently used [19].

i. We investigated the probability of each epoch being classified as REM sleep based on the preceding vigilance states. We found that if there are ten or more wake epochs among the twenty preceding an automatically identified REM sleep epoch, it is very unlikely to be REM sleep. Therefore, the entire REM sleep epoch is replaced with the state that immediately preceded it.
ii. Additionally, we *optionally* replace single REM sleep epochs with the preceding state: for example, NREM-NREM-*REM*-wake becomes NREM-NREM-*NREM*-wake.
iii. At this stage, we can finally reconstruct the states of artifact epochs. To maintain the initial number of epochs, those initially identified as artifacts are assigned to the same stage as the closest preceding non-artifact epoch. The visual examination of the EEG signal reveals that most artifacts occurred during wakefulness and very few during NREM and REM sleep, and the vigilance states of most of those epochs correspond to the vigilance state of the previous epoch. Thus, the continuity of the resulting hypnogram was not fragmented by artifacts. Artifact epochs were also included in the counting of the total amounts of each vigilance state. However, for the evaluation of EEG spectral power, all artifact epochs were excluded.

### 2.19. Second Iteration to Refine REM Sleep Detection

To further enhance the efficiency of REM sleep identification, a second iteration was conducted. This iteration employed the same clustering steps and application of contextual rules as the first iteration, with the key distinction being an updated preprocessing stage that removed additional wake and NREM epochs (Figure 4E,F). Such refinement allows for more precise identification of REM sleep during clustering and the application of contextual rules leading to a significant reduction in both false-positive and false-negative REM epochs compared to the first iteration in most animals. Particularly, the first iteration provides a rough estimation of epochs where REM sleep is possible. Given the parameters were set according to the recommended default values, the first iteration provides detection of nearly all REM sleep epochs at the cost of multiple false positive REM sleep epochs. To improve the results, we further eliminate epochs between the detected REM sleep epochs and the threshold. This step usually additionally eliminates hundreds of points, significantly changes the shape of each BC, and further reduces the overlap between wake and REM sleep epochs (Figure 4E,F). Thus, the second iteration provides improved results for REM sleep detection, mostly due to significant suppression of false positive REM sleep epochs.

**Figure 7:**
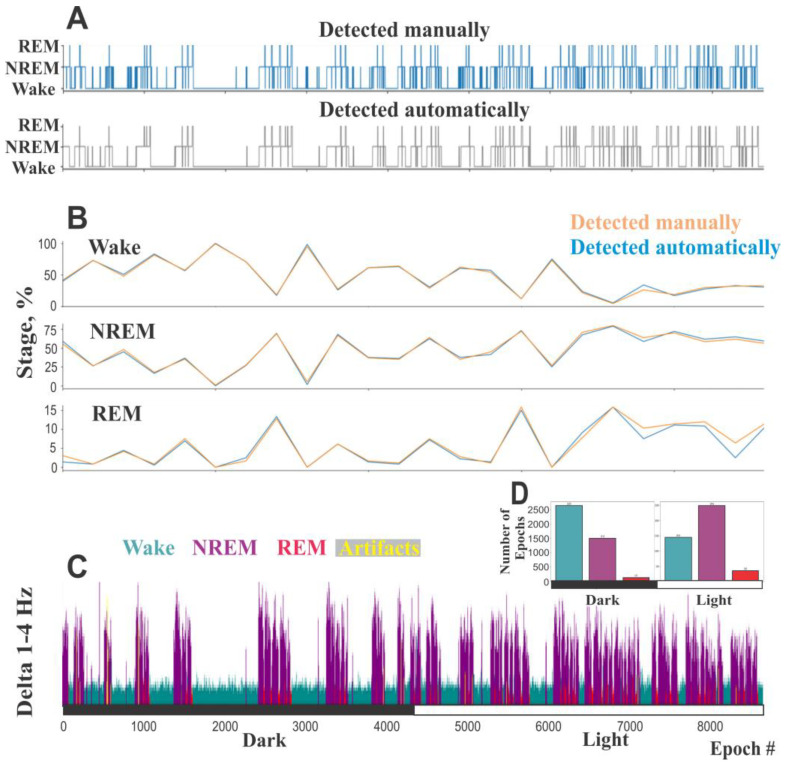
The figure illustrates the comparison of hypnograms and state percentages between automated and manual vigilance state identification, along with a graphical representation of the results from automatic sleep staging. (**A**). An exemplary hypnogram of mouse EEGs recorded over 24 h, generated by the automated algorithm (top) and the corresponding hypnogram derived from expert manual scoring (bottom). (**B**). Wake, NREM sleep, and REM sleep percentages calculated for the same recording using results from automatic EEG scoring (blue) and manual EEG+EMG scoring (orange) determined for each one-hour period. (**C**). Spectral power within the 1–4 Hz delta range, calculated for 10-s epochs from the same EEG dataset. Segments highlighted in green, purple, red, and yellow correspond to automatically identified states of wakefulness, NREM sleep, REM sleep, and artifacts, respectively. (**D**). The number of epochs classified as wakefulness, NREM sleep, and REM sleep during the dark and light periods. Artifact epochs were converted to the most likely corresponding vigilance states based on contextual criteria.

After this step, the final hypnogram is generated (Figure 7A).

### 2.20. Complementary Hypnogram Analysis

Once the hypnogram is obtained, the following analyses can be performed and compared with the expert data if necessary:

i. Stage percentage over a given window length (Figure 7B);
ii. Stage fragmentation: statistics on the length of continuous epochs for each stage saved as a .csv file;
iii. Power spectra for predefined frequency ranges color-coded according to automatically defined vigilance states (Figure 7C).
iv. Amount of each state for any chosen period of time (Figure 7D).

### 2.21. Checking the Scoring Quality

We scored the EEGs of 10 rats and 10 mice into three vigilance states using both the proposed automated algorithm and manual scoring, and we evaluated scoring quality by comparing the resulting automatic hypnograms to the expert-generated hypnogram. The evaluation criteria for scoring quality included multiple metrics: general accuracy, balanced accuracy, F1, Cohen’s kappa, accuracy by classes [7,39,40,41]. These metrics give a comprehensive insight into the performance of the automated scoring algorithm, ensuring a robust assessment of its ability to classify vigilance states accurately and consistently compared to manual expert scoring.

The Python implementation of the algorithm and accompanying instructions can be accessed at https://github.com/sykesva/SAGER (accessed on 30 January 2025).

## 3. Results

### 3.1. Estimation of Sensitivity of Different Frequency Bands for Separating NREM Sleep from Mix-W/REM Sleep

**Figure 8:**
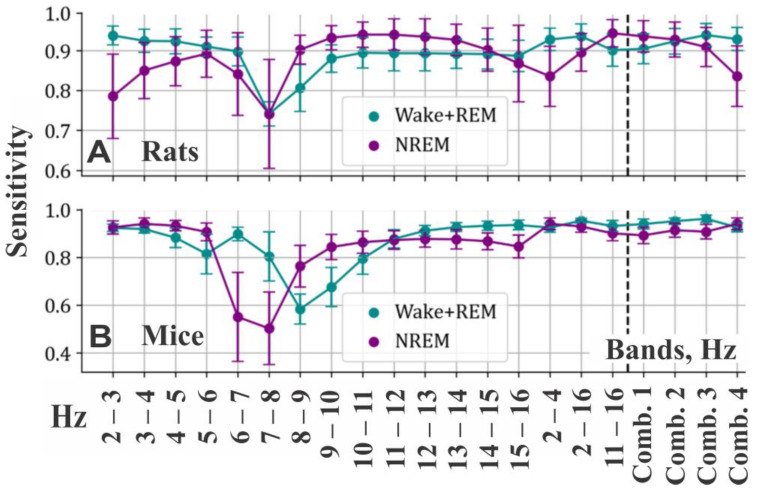
Sensitivity measures obtained in NREM vs rest classification averaged among 9 rats (**A**) and 10 mice (**B**); Comb. 1: 11–12, 12–13, 13–14, 14–15, 15–16 Hz; Comb. 2: 2–3, 3–4, 11–12, 12–13, 13–14, 14–15, 15–16 Hz; Comb. 3: 2–3, 3–4, 4–5, 5–6, 6–7, 7–8, 8–9, 9–10, 10–11, 11–12, 12–13, 13–14, 14–15, 15–16 Hz; Comb. 4: 2–3, 3–4, 2–4 Hz.

To effectively separate NREM sleep from mix-W/REM using GMM, we conducted a comprehensive analysis of classification quality across various frequency bands. We evaluated several metrics for both rats and mice during the initial NREM vs. mix-W/REM classification. Given that this is one of the early steps in the algorithm, we prioritized a “greedy” metric, such as sensitivity, which emphasizes true positive examples of the target class. This approach is warranted, as false positive NREM epochs can be corrected in subsequent steps (Figure 8).

The analysis revealed differences in sensitivities of the same bands between rats and mice (Figure 8). Therefore, we recommend selecting the most efficient bands for each species separately. Additionally, we propose that using combinations of bands yields superior and more robust sensitivities compared to relying on single bands.

Based on our analysis, we recommend using a single band of 11–16 Hz, along with band combinations 1, 2, and 3 (Figure 8) for rats. Combination 2 offers the best balance between sensitivities for NREM and mix-W/REM.

For mice, superior efficiency was achieved with single bands 2–3 Hz, 3–4 Hz, 2–4 Hz, 11–16 Hz, and 2–16 Hz, as well as with band combinations 2, 3, and 4 (Figure 8).

For this step of the automated sleep scoring algorithm, pure theta bands are unsurprisingly the least effective, while bands spanning delta and sigma ranges, as well as their combinations, prove to be the most useful.

### 3.2. Estimation of Sensitivity of Different BCs for Separating REM Sleep from Mix-W/NREM Sleep

We evaluated the ability of the BCs to retain the maximum number of REM sleep epochs within the REM sleep cluster while minimizing the inclusion of wake and NREM sleep epochs. To achieve this, we tested different combinations of theta and delta bands, including variations in band borders, widths, and numbers. This analysis encompassed all pairwise combinations of 15 theta bands within the 5–10 Hz range (OY bands) (with left borders from 5 to 9 Hz and width from 1 to 5 Hz) and 91 bands within the 2–16 Hz range (OX bands) (with left borders from 2 to 14 Hz and width from 2 to 14 Hz) (Figure 6A). For the 1365 resulting combinations, we performed the scoring up to step v of the “Final REM sleep identification” section and evaluated the REM sleep sensitivity (Figure 6A) based on binary REM sleep-vs-mix-W/NREM sleep classification. We selected a “greedy” metric that emphasizes true positive (TP) examples, as this is advantageous for the subsequent voting process. At this stage, it is important to generate as many TP REM sleep examples as possible, regardless of the false positive (FP) rate, since the latter will be reduced when combining results from different band combinations. The results are presented as heatmaps, with color-coded REM sleep sensitivity depending on the selected theta and delta/sigma bands (Figure 6A).

To determine the optimal EEG frequency bands for BCs necessary for efficient REM sleep classification, we systematically evaluated frequencies ranging from 2 to 16 Hz. We identified that, for rats, the most effective frequency bands were within the ranges of 5–10 Hz for the OY dimension and 2–7 Hz for the OX dimension. For mice, the optimal ranges were 5–10 Hz and 2–16 Hz, respectively.

To optimize REM sleep classification accuracy, it is advisable to avoid OY dimension bands that either exclude 7 Hz or have 7 Hz at either boundary, as these bands exhibit lower sensitivity (Figure 6A). Instead, selecting bands where 7 Hz is centrally positioned enhances classification performance (Figure 6A).

### 3.3. Estimation of Optimal Number of BCs and Stringency Thresholds for Different Frequency Bands for Separating REM Sleep from Mix-W/NREM Sleep

We also investigated how the scoring quality depends on the number of included BCs and the stringency of REM sleep identification (the number of BCs identifying the epoch as REM sleep) in both rats and mice (Supplementary Figure S2). For this purpose, only the most efficient BCs were selected according to their efficiency ranking. Since this is one of the final steps in the proposed sleep scoring approach, we considered comprehensive classification metrics—such as accuracy and F1 score across all stages (classes)—as criteria for evaluating scoring efficiency, given that these are commonly used metrics in classification problems. Additionally, we focused on the F1 score for REM sleep, treating it as a binary REM sleep-vs-mix-W/NREM sleep classification, since this analysis step primarily addresses REM sleep identification. Our goal was to simultaneously minimize and balance false positive and false negative REM sleep epochs by defining the optimal stringency threshold.

A visual assessment of averaged GMMs constructed with varying numbers of BCs revealed that increasing the number of BCs results in better isolation of the REM sleep cluster from wake and NREM clusters. This improves separation and enhances the accurate identification of REM sleep epochs (Figure 6B).

The results demonstrate that in rats, using even small numbers of BCs (≥4) provides the most accurate results, whereas in mice achieving the most accurate results requires using ≥15 BCs. The optimal stringency is ≥50% (more than half of the total number of BCs) for both species.

The step described above could be final. However, we designed more steps for the data processing to further improve the quality of the results.

### 3.4. Second Iteration

The implementation of the second iteration yielded improved results across all scoring quality metrics compared to the first iteration in most animals (Table 1).

### 3.5. Evaluating the Quality of Automatic Annotation in Rat and Mouse EEG Using Empirically Found Optimal Settings

The parameter settings for automatic scoring differed between rat and mouse EEGs. However, within each species group, identical parameters were applied to all animals. We scored the EEGs of 10 rats and 10 mice into three vigilance states using both the suggested automated algorithm and manual scoring. Setting parameters for automatic scoring of rat EEGs differed from parameters for mouse EEG. However, for scoring EEG within the group, parameters were identical for each animal.

For rats, the algorithm achieved a total accuracy of 0.92 and a balanced accuracy of 0.91. The accuracies for wake, NREM sleep, and REM sleep were 0.92, 0.92, and 0.91, respectively. The F1 scores for these states were 0.92, 0.91, and 0.89, while Cohen’s kappa values were 0.86, 0.85, and 0.87, respectively (Table 1).

In mice, using identical parameters, the algorithm achieved a total accuracy of 0.93 and a balanced accuracy of 0.92 (Table 1). The accuracies for wake, NREM sleep, and REM sleep were 0.94, 0.93, and 0.89, respectively. The F1 scores for these states were 0.93, 0.93, and 0.88, with Cohen’s kappa values of 0.87, 0.87, and 0.87, respectively (Table 1). By adjusting certain parameters, such as increasing the “ellipse size” (Figure 6C), we were able to improve the accuracy for REM sleep in mice to 0.91. However, this adjustment caused a slight decrease in wake accuracy from 0.94 to 0.93 due to the misclassification of wake epochs as false-positive REM sleep epochs.

Although we successfully conducted fully automated EEG analysis for all animals that are involved in our study (ten rats and ten mice), in some animals from specific groups the automatically delineated area within the REM sleep cluster was inadequately small. This issue resulted from a lower number of points identified as REM sleep epochs within the cluster. As a result, the small size of the delineated area was insufficient to reliably convert the most false-negative REM sleep epochs into true REM sleep epochs.

This inadequate delineation can be easily identified visually, as REM sleep clusters are typically distinct and easily recognizable. In such cases, the user may need to switch to manual mode to adjust the delineation. However, this adjustment requires only a few seconds and does not significantly delay the overall analysis, making it a minor inconvenience in an otherwise efficient process.

## 4. Discussion

The objective of this study was to develop and validate a Python-based algorithm for the automatic classification of vigilance states using single-channel EEG in rats and mice, two rodent species commonly used in sleep research. To achieve accuracy comparable to manual scoring of EEG and EMG polysomnographic recordings, we developed an original approach that integrates traditional clustering with novel preprocessing steps and species-specific EEG frequency band optimizations.

The key distinctive features of our algorithm include the following:

### 4.1. Artifact Preprocessing

The algorithm identifies both high-amplitude and normal-amplitude artifacts. The latter is achieved using a kernel density approach applied in the feature space of raw signal RMS and FFT amplitude. Epochs contaminated with artifacts are excluded from subsequent EEG analyses and are classified according to contextual rules after the first hypnogram is generated.

### 4.2. Clustering-Based Approach

All key steps are GMM-based or employ GMM, which effectively breaks the problem into simpler subtasks, enabling a more nuanced separation of vigilance states. This method is particularly effective at distinguishing NREM sleep from the mix of wake and REM sleep states, as well as in accurately identifying REM sleep.

### 4.3. Multi-Band Analysis for NREM Sleep and REM Sleep Identification

Unlike existing methods that typically rely on broad-band analyses, our approach utilizes narrow-band combinations. Systematic examination of the effectiveness of multiple delta and sigma frequency bands identified the optimal narrow bands and their combinations for a reliable sleep–wake state classification in both species. Multiband analysis tailored to the species-specific EEG characteristics in conjunction with a voting procedure across multiple bands are important methodological innovations enabling a reliable identification of NREM and REM sleep, as well as precise separation of REM sleep from wakefulness.

### 4.4. Complex Multi-Step REM Sleep Identification Process

*A* novel EEG preprocessing step, aimed at significantly reducing wake epochs, optimizes the subsequent clustering procedure and is crucial for improving the distinction between REM sleep and wakefulness. The use of a stringency-based “voting” procedure across multiple frequency bands further increases the accuracy of REM sleep identification.

### 4.5. Correction of False Negative REM Sleep Epochs

The algorithm features a novel geometry-based correction method that reassigns NREM sleep and wake epochs located deep within the REM sleep cluster to REM sleep. This step, along with contextual rules for refining the final hypnogram, eliminates physiologically implausible sequences and improves overall accuracy.

### 4.6. Comparing the Effectiveness of Our Algorithm with Existing Scoring Methods

Supplementary Table S1 summarizes the performance of ten recently published methods for scoring rodent PSG recordings. All studies reported performance that was comparable to or exceeded manual scoring. Approaches using both EEG and EMG generally outperformed single-EEG methods [42,43,44]. However, in cases where they specifically compared two methods, the difference was minimal (Supplementary Table S1), highlighting the feasibility of reliable sleep–wake staging using a single EEG channel.

Our approach achieves 93% accuracy for mice, which is comparable to, or slightly below, recent state-of-the-art studies that reported accuracies between 93% and 97% for mouse EEG [9,20,21,41,42,43]. Our REM sleep identification accuracy of 88% for mice closely aligns with the reported range of 85–90%. For rat EEGs, our overall accuracy of 92% matches the reported performance for rats (92%) [45]. While some studies reported slightly higher performance metrics than ours or others, this does not necessarily imply a definitive performance superiority of those methods. There is no standardized way for evaluating performance, and metrics may vary across studies due to differences in dataset composition, animal selection criteria, the number of expert scorers involved in generating ground truth annotations, and the experience level of each scorer. These methodological differences complicate direct comparisons between approaches.

For example, our overall accuracy for mice is slightly lower than the 97% reported by Brodersen [42], a difference likely influenced by contextual variations in ground truth labeling methodologies. In their study [46], the authors utilized annotations from multiple expert scorers and reconciled them into a consensus. This approach reduces individual biases, particularly for transitional states, by standardizing the dataset and producing a cleaner, more objective training set. As a result, this refinement improves the algorithm’s performance metrics by ensuring consistency in ground truth labels [42,46]. In contrast, in our study PSGs were scored by a single expert, which could introduce subjective biases and lower the apparent accuracy when compared to an idealized ground truth. Therefore, the slightly lower accuracy of our algorithm may reflect limitations in ground truth consistency rather than inherent shortcomings of the algorithm itself.

Moreover, comparisons with deep learning-based methods may be affected if these models analyze the same EEG dataset for both training and validation, as their performance is typically higher when applied to familiar data compared to an unfamiliar dataset.

The advantages of our algorithm include the following:

### 4.7. Transparency and Simplicity

Our algorithm integrates traditional signal processing, statistical analysis, and clustering techniques. The design ensures that each step is interpretable, and clearly understood, unlike in more complex machine learning models, such as deep learning neural networks, which can be opaque and require extensive training datasets.

### 4.8. Generalizability and Cross-Species Validation

Another strength of our algorithm is its validation across two rodent species, mice (C57BL/6) and rats (Fischer344), demonstrating consistently high accuracy (92–93%) for sleep–wake state classification. Many previous studies have focused primarily on either mice or rats, limiting the applicability of their findings to broader research contexts. By optimizing species-specific EEG frequency bands and testing the algorithm under various experimental conditions (spontaneous sleep, sleep deprivation, and recovery), we confirm the method’s robustness and generalizability to different datasets.

### 4.9. Reproducibility and Customizability

To ensure full reproducibility, our algorithm is implemented in a Python-based Jupyter Notebook with customizable parameters, allowing users to fine-tune EEG preprocessing, clustering criteria, and frequency band selection. The open-source availability of the code enables researchers to adapt the algorithm to new datasets and experimental conditions without requiring extensive retraining, as needed for deep learning approaches.

Additionally, the algorithm is designed for computational reproducibility by utilizing well-established scientific libraries such as MNE, SciPy, scikit-learn, and pandas. It also supports multiple EEG file formats through the Neo library, ensuring compatibility with various datasets. These features make the tool user-friendly and accessible to researchers, including those with limited programming experience. Furthermore, our algorithm is computationally efficient, allowing it to run on standard computers. It can process a full day of single-channel EEG data within 3–5 min on a PC equipped with an Intel i3 or i5 processor and 8 GB of RAM.

### 4.10. Potential for Optimization

The method’s flexibility and parameter-rich design provide significant opportunities for performance enhancement through further optimization. While this study explored only a limited subset of these options, the potential for users to identify and implement additional, more effective parameters during practical application is considerable. We provide the table of the main parameters that were used in our study (Supplementary Table S2). However, we encourage users to familiarize themselves with the method and actively experiment with adjusting the parameters to optimize and tailor the approach to their specific practical needs.

Further improvements in vigilance state identification could potentially be achieved by incorporating the beta and gamma EEG frequency ranges (15–30 Hz and 40–80 Hz, respectively) into the analysis, alongside the delta, theta, and sigma ranges [47,48,49,50], which would lead to significant gains in EEG scoring accuracy.

### 4.11. Limitations

i. The approach was tested on a limited number of mice and rats of different ages and genders under standard experimental conditions, including spontaneous sleep–wake cycles in undisturbed conditions and recovery sleep following sleep deprivation. While we expect comparable outcomes, further validation should involve rodents subjected to drug administration, chemo-genetic and optogenetic stimulation/inhibition, and transgenic animals. We acknowledge that pharmacological or genetically induced disturbances in the normal EEG pattern may affect sleep staging accuracy, requiring specific adaptations for reliable analysis.
ii. The performance of our method in handling heavily artifact-contaminated EEG recordings was not specifically evaluated. In the animals selected for this study, artifact contamination was low, affecting only 2–4% of all epochs. The affected epochs were assigned the same vigilance state as the preceding uncontaminated epoch. However, we anticipate that with heavier contamination, the performance of the artifact processing may be limited, as the loss of signal continuity could obscure vigilance state classification.
iii. Reliance on a single expert scorer introduces potential biases in the ground truth annotations, as subjective variability in interpreting ambiguous epochs may affect the consistency and accuracy of the expert data, which is crucial for empirically defining the rules utilized in subsequent automatic scoring.
iv. In some instances, the automatic delineation of the REM sleep cluster may not be optimal, requiring manual intervention by the user. However, this adjustment is straightforward, time-efficient, and does not substantially impact the overall analysis workflow.
v. Although our approach achieves REM sleep staging performance comparable to manual scoring and state-of-the-art methods, a challenge remains in the misclassification of a small fraction of transitional sleep–wake states. While this minor misclassification is unlikely to significantly affect most sleep studies, further development of more refined methods is needed to minimize this limitation.

## 5. Conclusions

In this study, we developed and validated an open-source Python-based algorithm for the automatic staging of vigilance states in rodents using single EEG signals. The algorithm demonstrates performance comparable to state-of-the-art methods reported in recent studies. Our approach offers insights into distinguishing REM sleep from wake states, a main challenge in vigilance state classification based on EEG analysis only.

Key strengths of the algorithm include integration of original methods of EEG preprocessing, species-specific frequency band optimizations, and a multi-step clustering for accurate identification of sleep–wake states. A detailed examination of delta, theta, and sigma frequency ranges revealed optimal frequency bands and their combinations for rats and mice, enhancing the reliability of state identification. The use of GMM facilitated the effective separation of wakefulness, NREM, and REM sleep, while the application of contextual rules further enhanced the accuracy of the final hypnograms.

The algorithm proved to be time-efficient and adaptable for bulk data analysis, making it a reliable tool for longitudinal and large-scale sleep studies in rodents. It represents a step forward in automated sleep staging in rodents and can be further adapted for human research and different laboratory species, contributing to both methodological and scientific advancements in sleep studies.

## Figures and Tables

**Table 1:** Animal dataset and performance metrics of our approach tested on two species after the first and second iterations.

Rats					Iteration 1													Iteration 2												
					Ellipse Size 1.8													Ellipse Size 1.2												
					Accuracy by Class			General Accuracy	Balanced Accuracy	F1 by Class			General F1	Cohen’s Kappa by Class			General Cohen’s Kappa	Accuracy by Class			General Accuracy	Balanced Accuracy	F1 by Class			General F1	Cohen’s kappas by class:			General Cohen’s kappa
#	Age (m)	Gen- der	Condition	Duration (h)	W	NR	R			W	NR	R		W	NR	R		W	NR	R			W	NR	R		W	NR	R	
1	4	m	6h SD	24	0.93	0.88	0.97	0.91	0.93	0.94	0.91	0.84	0.92	0.88	0.85	0.81	0.86	0.93	0.92	0.94	0.93	0.93	0.94	0.92	0.91	0.93	0.89	0.87	0.89	0.88
2	4	m	6h SD	24	0.91	0.93	0.91	0.92	0.92	0.93	0.91	0.88	0.92	0.86	0.85	0.87	0.86	0.91	0.94	0.88	0.92	0.91	0.93	0.91	0.89	0.92	0.86	0.85	0.88	0.86
3	4	m	BL	24	0.90	0.92	0.83	0.90	0.88	0.91	0.90	0.84	0.90	0.83	0.83	0.83	0.83	0.91	0.93	0.83	0.91	0.89	0.92	0.91	0.87	0.91	0.84	0.84	0.85	0.84
4	4	m	6h SD	24	0.92	0.87	0.91	0.90	0.90	0.92	0.89	0.83	0.90	0.83	0.83	0.81	0.83	0.92	0.92	0.93	0.92	0.92	0.94	0.90	0.92	0.92	0.86	0.85	0.91	0.86
5	12	m	6h SD	24	0.93	0.91	0.98	0.93	0.94	0.95	0.91	0.87	0.93	0.90	0.86	0.85	0.88	0.94	0.93	0.96	0.94	0.94	0.95	0.92	0.91	0.94	0.90	0.87	0.91	0.89
6	12	m	6h SD	24	0.87	0.88	0.93	0.88	0.89	0.91	0.90	0.65	0.89	0.83	0.84	0.61	0.80	0.93	0.93	0.90	0.93	0.92	0.94	0.92	0.86	0.93	0.88	0.87	0.85	0.87
7	6	m	6h SD	24	0.88	0.85	0.96	0.88	0.90	0.91	0.89	0.71	0.88	0.81	0.83	0.67	0.79	0.94	0.90	0.88	0.92	0.91	0.94	0.91	0.86	0.92	0.87	0.86	0.84	0.86
8	6	m	Recovery	16	0.87	0.85	0.95	0.87	0.89	0.86	0.89	0.82	0.87	0.80	0.77	0.79	0.78	0.89	0.89	0.91	0.90	0.90	0.86	0.91	0.92	0.90	0.80	0.81	0.90	0.82
9	6	m	Recovery	16	0.90	0.86	0.91	0.88	0.89	0.90	0.91	0.78	0.89	0.85	0.83	0.73	0.81	0.92	0.91	0.84	0.90	0.89	0.90	0.92	0.84	0.90	0.84	0.86	0.81	0.84
10	6	m	Recovery	16	0.86	0.92	0.95	0.90	0.91	0.90	0.92	0.85	0.90	0.83	0.84	0.83	0.84	0.87	0.93	0.93	0.91	0.91	0.90	0.92	0.89	0.91	0.84	0.84	0.88	0.85
aver					0.90	0.88	0.94	0.90	0.91	0.91	0.90	0.80	0.90	0.84	0.83	0.77	0.83	0.92	0.92	0.91	0.92	0.91	0.92	0.91	0.89	0.92	0.86	0.85	0.87	0.86
Mice					Iteration_1													Iteration_2												
					Ellipse Size_1.8													Ellipse Size_1.8												
					Accuracy by Class			General Accuracy	Balanced Accuracy	F1 by Class			General F1	Cohen’s Kappas by Class:			General Cohen’s Kappa	Accuracy by Class			General Accuracy	Balanced Accuracy	F1 by Class			General F1	Cohen’s Kappas by Class:			General Cohen’s Kappa
#	Age (m)	Gen- der	Condition	Duration (h)	W	NR	R			W	NR	R		W	NR	R		W	NR	R			W	NR	R		W	NR	R	
1	25	m	BL	24	0.90	0.93	0.91	0.92	0.92	0.92	0.93	0.80	0.92	0.86	0.85	0.78	0.85	0.91	0.94	0.96	0.93	0.94	0.92	0.94	0.87	0.93	0.88	0.87	0.86	0.87
2	25	m	BL	24	0.94	0.91	0.88	0.92	0.91	0.93	0.92	0.86	0.92	0.86	0.86	0.85	0.86	0.94	0.91	0.92	0.92	0.92	0.93	0.92	0.89	0.92	0.86	0.86	0.88	0.86
3	25	m	BL	24	0.94	0.91	0.90	0.92	0.91	0.92	0.94	0.80	0.92	0.86	0.87	0.78	0.86	0.94	0.91	0.89	0.92	0.91	0.92	0.93	0.83	0.92	0.86	0.87	0.82	0.86
4	25	m	BL	24	0.91	0.89	0.85	0.89	0.88	0.86	0.92	0.86	0.90	0.79	0.80	0.85	0.80	0.91	0.89	0.87	0.90	0.89	0.87	0.92	0.88	0.90	0.79	0.80	0.88	0.80
5	25	m	BL	24	0.93	0.91	0.89	0.92	0.91	0.91	0.93	0.84	0.92	0.85	0.85	0.83	0.85	0.93	0.91	0.90	0.92	0.92	0.91	0.93	0.86	0.92	0.86	0.85	0.85	0.85
6	4	f	BL	24	0.92	0.95	0.87	0.93	0.91	0.94	0.94	0.86	0.93	0.89	0.88	0.85	0.88	0.93	0.96	0.83	0.94	0.91	0.94	0.94	0.87	0.93	0.89	0.88	0.86	0.88
7	4	f	BL	24	0.95	0.94	0.82	0.94	0.90	0.95	0.94	0.88	0.94	0.89	0.89	0.87	0.89	0.95	0.94	0.87	0.94	0.92	0.95	0.94	0.91	0.94	0.90	0.89	0.91	0.89
8	4	f	BL	24	0.95	0.96	0.80	0.95	0.90	0.96	0.94	0.86	0.95	0.91	0.90	0.86	0.90	0.95	0.96	0.81	0.95	0.91	0.96	0.94	0.87	0.95	0.91	0.90	0.86	0.90
9	3	f	BL	24	0.95	0.93	0.85	0.93	0.91	0.94	0.94	0.88	0.93	0.88	0.88	0.87	0.88	0.94	0.94	0.90	0.94	0.93	0.94	0.94	0.90	0.94	0.89	0.88	0.90	0.89
10	3	f	BL	24	0.91	0.94	0.91	0.92	0.92	0.92	0.93	0.87	0.92	0.86	0.86	0.86	0.86	0.92	0.94	0.90	0.93	0.92	0.93	0.94	0.89	0.93	0.87	0.86	0.89	0.87
aver					0.93	0.93	0.87	0.92	0.91	0.93	0.93	0.85	0.93	0.87	0.86	0.84	0.86	0.93	0.93	0.89	0.93	0.92	0.93	0.93	0.88	0.93	0.87	0.87	0.87	0.87

**Table 2:** Delta/sigma and theta bands for BC construction for REM sleep identification.

Frequency Range	Band, Hz
Delta/sigma	2–4, 2–5, 2–6, 2–7, 2–8, 2–9, 2–10, 2–11, 2–12, 2–13, 2–14, 2–15, 2–16, 3–5, 3–6, 3–7, 3–8, 3–9, 3–10, 3–11, 3–12, 3–13, 3–14, 3–15, 3–16, 4–6, 4–7, 4–8, 4–9, 4–10, 4–11, 4–12, 4–13, 4–14, 4–15, 4–16, 5–7, 5–8, 5–9, 5–10, 5–11, 5–12, 5–13, 5–14, 5–15, 5–16, 6–8, 6–9, 6–10, 6–11, 6–12, 6–13, 6–14, 6–15, 6–16, 7–9, 7–10, 7–11, 7–12, 7–13, 7–14, 7–15, 7–16, 8–10, 8–11, 8–12, 8–13, 8–14, 8–15, 8–16, 9–11, 9–12, 9–13, 9–14, 9–15, 9–16, 10–12, 10–13, 10–14, 10–15, 10–16, 11–13, 11–14, 11–15, 11–16, 12–14, 12–15, 12–16, 13–15, 13–16, 14–16
Theta	5–6, 5–7, 5–8, 5–9, 5–10, 6–7, 6–8, 6–9, 6–10, 7–8, 7–9, 7–10, 8–9, 8–10, 9–10

## Data Availability

The dataset sufficiently describes the data. The code designed for this study is openly accessible via the link: https://github.com/sykesva/SAGER (accessed on 30 January 2025).

## Supplementary Materials

The following supporting information can be downloaded at: https://www.mdpi.com/article/10.3390/s25030921/s1.

## Abbreviations

The following abbreviations are used in this manuscript:

GMM	Gaussian mixture model
EEG	Electroencephalogram
NREM	Non-rapid eye movement sleep
REM	Rapid eye movement sleep
W	Wake
PSG	Polysomnographic
ANN	Artificial neural networks
RMS	Root mean square
FFT	Fast Fourier transform
BC	Band combination

## References

[1] Ambrogio C., Koebnick J., Quan S.F., Ranieri V.M., Parthasarathy S. (2008). Assessment of Sleep in Ventilator-Supported Critically Ill Patients. Sleep.

[2] Danker-Hopfe H., Kunz D., Gruber G., Klösch G., Lorenzo J.L., Himanen S.L., Kemp B., Penzel T., Röschke J., Dorn H. (2004). Interrater reliability between scorers from eight European sleep laboratories in subjects with different sleep disorders. J. Sleep Res..

[3] Hsieh K.-C., Robinson E.L., Fuller C.A. (2008). Sleep Architecture in Unrestrained Rhesus Monkeys (*Macaca mulatta*) Synchronized to 24-Hour Light-Dark Cycles. Sleep.

[4] Louis R.P., Lee J., Stephenson R. (2004). Design and validation of a computer-based sleep-scoring algorithm. J. Neurosci. Methods.

[5] Costa-Miserachs D., Portell-Cortés I., Torras-Garcia M., Morgado-Bernal I. (2003). Automated sleep staging in rat with a standard spreadsheet. J. Neurosci. Methods.

[6] Rayan A., Agarwal A., Samanta A., Severijnen E., van der Meij J., Genzel L. (2022). Sleep scoring in rodents: Criteria, automatic approaches and outstanding issues. Eur. J. Neurosci..

[7] Fang G., Xia Y., Zhang C., Liu T., Yao D. (2010). Optimized single electroencephalogram channel sleep staging in rats. Lab. Anim..

[8] Tezuka T., Kumar D., Singh S., Koyanagi I., Naoi T., Sakaguchi M. (2021). Real-time, automatic, open-source sleep stage classification system using single EEG for mice. Sci. Rep..

[9] Rahimi S., Soleymankhani A., Joyce L., Matulewicz P., Kreuzer M., Fenzl T., Drexel M. (2023). Discriminating rapid eye movement sleep from wakefulness by analyzing high frequencies from single-channel EEG recordings in mice. Sci. Rep..

[10] Lin N.-H., Hsu C.-Y., Luo Y., Nagurka M.L., Sung J.-L., Hong C.-Y., Yen C.-W. (2017). Detecting rapid eye movement sleep using a single EEG signal channel. Expert Syst. Appl..

[11] Rytkönen K.-M., Zitting J., Porkka-Heiskanen T. (2011). Automated sleep scoring in rats and mice using the naive Bayes classifier. J. Neurosci. Methods.

[12] Fenzl T., Romanowski CP N., Flachskamm C., Honsberg K., Boll E., Hoehne A., Kimura M. (2007). Fully automated sleep deprivation in mice as a tool in sleep research. J. Neurosci. Methods.

[13] Zhu M., Chen J., Li H., Liang F., Han L., Zhang Z. (2021). Vehicle driver drowsiness detection method using wearable EEG based on convolution neural network. Neural Comput. Appl..

[14] Tortora S., Ghidoni S., Chisari C., Micera S., Artoni F. (2020). Deep learning-based BCI for gait decoding from EEG with LSTM recurrent neural network. J. Neural Eng..

[15] Matsumori S., Teramoto K., Iyori H., Soda T., Yoshimoto S., Mizutani H. (2022). HARU Sleep: A Deep Learning-Based Sleep Scoring System with Wearable Sheet-Type Frontal EEG Sensors. IEEE Access.

[16] Liu Y., Yang Z., You Y., Shan W., Ban W. (2022). An attention-based temporal convolutional network for rodent sleep stage classification across species, mutants and experimental environments with single-channel electroencephalogram. Physiol. Meas..

[17] Navas-Olive A., Amaducci R., Jurado-Parras M.-T., Sebastian E.R., de la Prida L.M. (2022). Deep learning-based feature extraction for prediction and interpretation of sharp-wave ripples in the rodent hippocampus. eLife.

[18] Sunagawa G.A., Séi H., Shimba S., Urade Y., Ueda H.R. (2013). FASTER: An unsupervised fully automated sleep staging method for mice. Genes Cells.

[19] Lacroix M.M., de Lavilléon G., Lefort J., El Kanbi K., Bagur S., Laventure S., Dauvilliers Y., Peyron C., Benchenane K. (2018). Improved sleep scoring in mice reveals human-like stages. bioRxiv.

[20] Cusinato R., Gross S., Bainier M., Janz P., Schoenenberger P., Redondo R.L. (2024). Workflow for the unsupervised clustering of sleep stages identifies light and deep sleep in electrophysiological recordings in mice. J. Neurosci. Methods.

[21] Koyanagi I., Tezuka T., Yu J., Srinivasan S., Naoi T., Yasugaki S., Nakai A., Taniguchi S., Hayashi Y., Nakano Y. (2023). Fully automatic REM sleep stage-specific intervention systems using single EEG in mice. Neurosci. Res..

[22] Kostin A., Alam Md A., Siegel J.M., McGinty D., Alam M.N. (2020). Sex- and Age-dependent Differences in Sleep-wake Characteristics of Fisher-344 Rats. Neuroscience.

[23] Kostin A., Alam Md A., Saevskiy A., Alam M.N. (2024). Chronic Astrocytic TNFα Production in the Preoptic-Basal Forebrain Causes Aging-like Sleep–Wake Disturbances in Young Mice. Cells.

[24] Alam M.A., Mallick B.N. (2008). Glutamic acid stimulation of the perifornical-lateral hypothalamic area promotes arousal and inhibits non-REM/REM sleep. Neurosci. Lett..

[25] Kostin A., Alam Md A., McGinty D., Szymusiak R., Alam M.N. (2019). Chronic Suppression of Hypothalamic Cell Proliferation and Neurogenesis Induces Aging-Like Changes in Sleep–Wake Organization in Young Mice. Neuroscience.

[26] Kostin A., Siegel J.M., Alam M.N. (2014). Lack of Hypocretin Attenuates Behavioral Changes Produced by Glutamatergic Activation of the Perifornical-Lateral Hypothalamic Area. Sleep.

[27] Granger B.E., Perez F. (2021). Jupyter: Thinking and Storytelling with Code and Data. Comput. Sci. Eng..

[28] Garcia S., Guarino D., Jaillet F., Jennings T., Pröpper R., Rautenberg P.L., Rodgers C.C., Sobolev A., Wachtler T., Yger P. (2014). Neo: An object model for handling electrophysiology data in multiple formats. Front. Neuroinform..

[29] Larson E., Gramfort A., Engemann D.A., Leppakangas J., Brodbeck C., Jas M., Brooks T.L., Sassenhagen J., McCloy D., Luessi M. (2024). MNE-Python (Version v1.8.0). Zenodo.

[30] Gramfort A. (2013). MEG and EEG data analysis with MNE-Python. Front. Neurosci..

[31] Kreuzer M., Polta S., Gapp J., Schuler C., Kochs E.F., Fenzl T. (2015). Sleep scoring made easy—Semi-automated sleep analysis software and manual rescoring tools for basic sleep research in mice. MethodsX.

[32] Durán E., Oyanedel C.N., Niethard N., Inostroza M., Born J. (2018). Sleep stage dynamics in neocortex and hippocampus. Sleep.

[33] Pedregosa F., Varoquaux G., Gramfort A., Michel V., Thirion B., Grisel O., Blondel M., Müller A., Nothman J., Louppe G. (2012). Scikit-learn: Machine Learning in Python. arXiv.

[34] Dempster A.P., Laird N.M., Rubin D.B. (1977). Maximum Likelihood from Incomplete Data via the EM Algorithm. J. R. Stat. Soc. (Ser. B).

[35] Mondino A., Cavelli M., Gonzalez J., Osorio L., Castro-Zaballa S., Costa A., Vanini G., Torterolo P. (2020). Power and coherence in the EEG of the rat: Impact of behavioral states, cortical area, lateralization and light/dark phases. Clocks Sleep.

[36] Kim D., Hwang E., Lee M., Sung H., Choi J.H. (2015). Characterization of Topographically Specific Sleep Spindles in Mice. Sleep.

[37] Jolliffe I. (2011). Principal Component Analysis. Int. Encycl. Stat. Sci..

[38] Rudiger P., Stevens J.-L., Hansen S.H., Bednar J.A., Liquet M., Andrew, Nijholt B., Mease J., Chris B., Randelhoff A. (2024). holoviz/holoviews: Version 1.19.1. Zenodo.

[39] Hossin M., Sulaiman M.N. (2015). A Review on Evaluation Metrics for Data Classification Evaluations. Int. J. Data Min. Knowl. Manag. Process.

[40] Ferrer L. (2022). Analysis and Comparison of Classification Metrics (Version 4). arXiv.

[41] Bakker J.P., Ross M., Cerny A., Vasko R., Shaw E., Kuna S., Magalang U.J., Punjabi N.M., Anderer P. (2022). Scoring sleep with artificial intelligence enables quantification of sleep stage ambiguity: Hypnodensity based on multiple expert scorers and auto-scoring. Sleep.

[42] Brodersen PJ N., Alfonsa H., Krone L.B., Blanco-Duque C., Fisk A.S., Flaherty S.J., Guillaumin MC C., Huang Y.-G., Kahn M.C., McKillop L.E. (2024). Somnotate: A probabilistic sleep stage classifier for studying vigilance state transitions. PLoS Comput. Biol..

[43] Schwabedal J.T.C., Sippel D., Brandt M.D., Bialonski S. (2018). Automated Classification of Sleep Stages and EEG Artifacts in Mice with Deep Learning (Version 1). arXiv.

[44] Wang L.A., Kern R., Yu E., Choi S., Pan J.Q. (2023). IntelliSleepScorer, a software package with a graphic user interface for automated sleep stage scoring in mice based on a light gradient boosting machine algorithm. Sci. Rep..

[45] Yamada R.G., Matsuzawa K., Ode K.L., Ueda H.R. (2024). An automated sleep staging tool based on simple statistical features of mice electroencephalography (EEG) and electromyography (EMG) data. Eur. J. Neurosci..

[46] Danker-Hopfe H., Anderer P., Zeitlhofer J., Boeck M., Dorn H., Gruber G., Heller E., Loretz E., Moser D., Parapatics S. (2009). Interrater reliability for sleep scoring according to the Rechtschaffen & Kales and the new AASM standard. J. Sleep Res..

[47] Bouyer J.J., Montaron M.F., Rougeul A. (1981). Fast fronto-parietal rhythms during combined focused attentive behaviour and immobility in cat: Cortical and thalamic localizations. Electroencephalogr. Clin. Neurophysiol..

[48] Jeantet Y., Cayzac S., Cho Y.H. (2013). β Oscillation during Slow Wave Sleep Rapid Eye Movement Sleep in the Electroencephalogram of a Transgenic Mouse Model of Huntington’s Disease. PLoS ONE.

[49] Grønli J., Rempe M.J., Clegern W.C., Schmidt M., Wisor J.P. (2016). Beta EEG reflects sensory processing in active wakefulness and homeostatic sleep drive in quiet wakefulness. J. Sleep Res..

[50] Brankačk J., Kukushka V.I., Vyssotski A.L., Draguhn A. (2010). EEG gamma frequency and sleep–wake scoring in mice: Comparing two types of supervised classifiers. Brain Res..

